# DockBench: An Integrated Informatic Platform Bridging the Gap between the Robust Validation of Docking Protocols and Virtual Screening Simulations

**DOI:** 10.3390/molecules20069977

**Published:** 2015-05-29

**Authors:** Alberto Cuzzolin, Mattia Sturlese, Ivana Malvacio, Antonella Ciancetta, Stefano Moro, Rino Ragno

**Affiliations:** 1 Molecular Modeling Section (MMS), Department of Pharmaceutical and Pharmacological Sciences, University of Padova, via Marzolo 5, Padova 35131, Italy; E-Mails: alberto.cuzzolin@studenti.unipd.it (A.Cu.); mattia.sturlese@unipd.it (M.S.); antonella.ciancetta@unipd.it (A.Ci.); 2 INFIQC—Organic Chemistry Department, School of Chemical Sciences, National University of Cordoba, Cordoba, CP 5000, Argentine; E-Mail: ivanamalvacio@hotmail.com

**Keywords:** molecular docking, *docking benchmark*, virtual screening, structure-based drug design

## Abstract

Virtual screening (VS) is a computational methodology that streamlines the drug discovery process by reducing costs and required resources through the *in silico* identification of potential drug candidates. Structure-based VS (SBVS) exploits knowledge about the three-dimensional (3D) structure of protein targets and uses the docking methodology as search engine for novel hits. The success of a SBVS campaign strongly depends upon the accuracy of the docking protocol used to select the candidates from large chemical libraries. The identification of suitable protocols is therefore a crucial step in the setup of SBVS experiments. Carrying out extensive benchmark studies, however, is usually a tangled task that requires users’ proficiency in handling different file formats and philosophies at the basis of the plethora of existing software packages. We present here DockBench 1.0, a platform available free of charge that eases the pipeline by automating the entire procedure, from docking benchmark to VS setups. In its current implementation, DockBench 1.0 handles seven docking software packages and offers the possibility to test up to seventeen different protocols. The main features of our platform are presented here and the results of the benchmark study of human Checkpoint kinase 1 (hChk1) are discussed as validation test.

## 1. Introduction

Virtual screening is a computational methodology aimed at streamlining the drug discovery process through the *in silico* identification of novel hits from large chemical libraries [1]. After emerging in the late 1990s [2] as a strategy to reduce the time and cost of chemical synthesis and *in vitro* testing, VS nowadays represents an integral part of the drug discovery pipeline both in industry and in academic environments [3]. The main purpose of a VS campaign is to select appropriate compounds while removing unsuitable structures, thus significantly reducing costs and required resources. Depending on the amount of information available about the system of interest, VS is historically classified into two main categories [4]: ligand-based VS (LBVS) and structure-based VS (SBVS). SBVS exploits knowledge about the three-dimensional (3D) structure of the target—gathered either experimentally by X-ray crystallography or NMR spectroscopy, or computationally through homology modelling—and performs docking calculations to rank candidates on the basis of estimated binding affinity or complementarity to the binding site [5].

Consequently, the success of a SBVS campaign strongly depends upon the accuracy of the engine used to generate, place, and rank the conformation of candidates into a target binding site [6]. A crucial step in the setup of a SBVS experiment is therefore the selection of a proper docking protocol, *i.e.*, the combination of search algorithm and scoring function that yields the best accuracy achievable.

The comparison of different docking protocols is not a trivial task [7], as it requires expertise in handling the different philosophies behind the large variety of available software packages. As a result, non-expert users are usually discouraged in enriching the pool of docking programs to test due to difficulties in input and output formats syntax comprehension, conversion and management. Moreover, the time required in merging and comparing the results arising from different protocols usually is incompatible with the requests of the experimental counterpart.

Within this framework, we have recently proposed [8] a strategy to compare the performances of docking protocols based on two quality metrics: The “Protocol Score”, and the number of conformations generated by the docking protocol with a RMSD value below the resolution (R) of the crystal structure “N^(RMSD < R)^”. With the aim to broaden their exploitation also by non-expert users, we have proposed the presentation of the results as coloured maps of immediate interpretation in a benchmark study focused on the human adenosine 2A receptor.

In the present work, we move a step forward and present DockBench 1.0, a platform available free of charge upon request that fully automates the entire procedure from the setup of docking benchmarks to VS campaigns. In its current implementation, DockBench 1.0 handles seven different docking software packages and provides the user with the possibility to test up to seventeen protocols. A GUI guides the user step-by-step throughout all the stages required to perform the entire pipeline, from the choice of the docking protocol to assess to the VS of large chemical libraries. The results are expressed in terms of the above mentioned quality metrics and returned as easy to interpret coloured maps. The outputs of the different software packages are returned in a unique format and are analysed with a standardized procedure to avoid software related biases.

We describe as validation case a docking benchmark study focused on human checkpoint kinase 1 (hChk1). hChk1 is a serine/threonine kinase responsible for the arrest of the cell cycle that allow DNA repair in tumour cells in response to a damage [9]. Therefore, hChk1 inhibition represents a strategy to increase the therapeutic efficacy of anticancer drugs, thus enhancing the apoptosis induced by alkylating agents [10,11].

## 2. Results and Discussion

### 2.1. DockBench General Features

**Figure 1:**
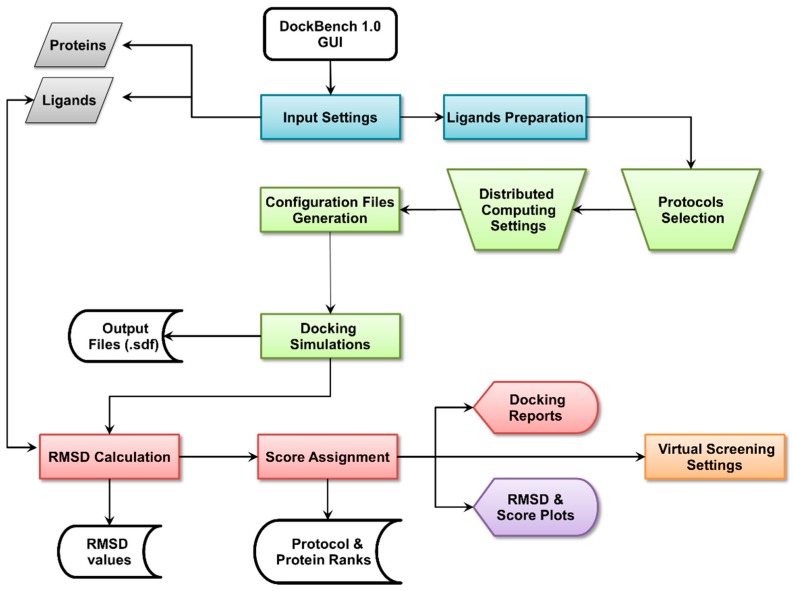
DockBech 1.0 workflow. The platform is accessed through a GUI, the different stages of the pipeline are highlighted with different colours.

**Figure 2:**
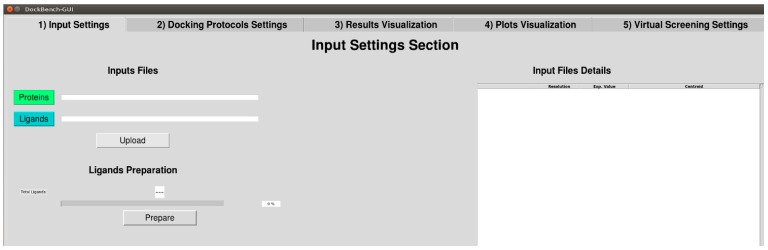
DockBench GUI tabs: (1) Input Settings; (2) Docking Protocols Settings; (3) Results Visualization; (4) Plots Visualization; (5) Virtual Screening Settings.

The Flowchart of the DockBench 1.0 platform is reported in Figure 1. All the functionalities are embedded in a graphical user interface (GUI, Figure 2) and are organized into five main tabs, corresponding to the tasks required to carry out a complete pipeline, from docking benchmark studies to VS experiments: (1) Input Settings; (2) Docking Protocols Settings; (3) Results Visualization; (4) Plots Visualization; (5) Virtual Screening Settings. The main features of each tab are discussed in the following. DockBench 1.0 is available free of charge and can be requested at the project page [12].

#### 2.1.1. Input Settings

DockBench 1.0 significantly eases benchmark and VS procedures and allows the user to submit jobs with the different implemented software packages at once. The user is asked to provide a few files including ligands and receptors structures in Tripos .mol2 format and receptor structures .pdb format as retrieved from the Protein Data Bank (PDB) [13] (Figure 1, grey boxes). The supplied structures, apart from original pdb files, must be prepared in advance. In particular, hydrogen atoms need to be added by setting the correct protonation states for both the ligand and the protein structures. Moreover, the user must take care of generating proper ligands tautomeric and stereoisomeric states. Once the structures have been uploaded, the R values, ligand names and pdb codes are automatically extracted from PDB Remark section, displayed in a table on the GUI and saved for subsequent use for files nomenclature and results visualization. Available binding data information for the co-crystallized ligands are directly retrieved from PDB source page and displayed. In case several data are available, the following selection hierarchy is applied: Ki, Kd, and IC_50_.

After the input structures have been uploaded, the coordinates of ligands centroids are computed according to Equation (1) and set as the binding cavity centre for the subsequent docking simulations. To avoid biases due to input conformations, a ligands preparation step (Figure 1, blue boxes) is performed with the obminimize [14] tool, as detailed in the Methods section.

#### 2.1.2. Docking Protocols Settings

In the protocol selection tab all the implemented docking software packages are listed. In case a docking program is not available to the user, it will be automatically set as inactive. DockBench 1.0 offers the possibility to select up to 17 different protocols, sorted alphabetically as reported in Table 1. Briefly, AutoDock [15] is embedded with three different global optimizer approaches coupled with the AutoDock Scoring Function: Genetic algorithm (GA), Lamarkian genetic algorithm (LGA), and local search (LS). AutoDock Vina [16] is included with its standard optimization algorithm and standard scoring function. Glide [17,18], is implemented with the Standard Precision mode. Four scoring functions are available for the GOLD suite (ASP, Chemscore, Goldscore and PLP) [19]. Plants [20] is available with three different scoring functions [21] (ChemPLP, PLP, PLP95) that are coupled to the Ant Colony Optimization (ACO) algorithm. The Triangle Matcher placing method of the MOE docking tool is implemented along with three different scoring functions (Affinity dG, London dG, GBVI/WSA) [22]. rDock [23] can be run with or without desolvation potential term with its standard scoring function. Each protocol is managed independently by providing the user with the possibility to select all of them or individual ones.

**Table 1:** List of docking protocols available in DockBench 1.0.

Program	Search Algorithm/Placing Method	Scoring Function	Protocol Abbreviation
Autodock 4.2	Local Search	AutoDock SF	AUTODOCK-ls
	Lamarkian GA	AutoDock SF	AUTODOCK-lga
	Genetic Algorithm	AutoDock SF	AUTODOCK-ga
AutoDock Vina 1.1.2	Monte Carlo + BFGS local search	Standard Vina SF	VINA-std
Glide 6.5	Glide Algorithm	Standard Precision	GLIDE-sp
GOLD 5.2	Genetic Algorithm	Goldscore	GOLD-goldscore
	Genetic Algorithm	Chemscore	GOLD-chemscore
	Genetic Algorithm	ASP	GOLD-asp
	Genetic Algorithm	PLP	GOLD-plp
MOE 2014.09	Triangle Matcher	London-dG	MOE-londondg
	Triangle Matcher	Affinity-dG	MOE-affinitydg
	Triangle Matcher	GBIVIWSA	MOE-gbiviwsa
PLANTS 1.2	ACO Algorithm	PLP	PLANTS-plp
	ACO Algorithm	PLP95	PLANTS-plp95
	ACO Algorithm	ChemPLP	PLANTS-chemplp
rDock 2013.1	Genetic Algorithm + Monte Carlo + Simplex minimization	Standard rDock master SF	RDOCK-std
	Genetic Algorithm + Monte Carlo + Simplex minimization	Standard rDock master SF + desolvation potential	RDOCK-solv

Several advanced options (Figure 1, green trapezoids) can be customized by the user prior to running the docking simulations: The number of output poses (default 20), the threshold RMSD value to define unique poses (default 1.0 Å, not available for AutoDock Vina and rDOCK), and the radius (default 20 Å) of the binding site. As several software packages describe the binding site using inclusion spheres (GOLD, PLANTS, rDOCK), the sphere radius *r* is set as common parameter to define the cavity. Nevertheless, to maintain comparable volumes for the protocols adopting parallelepiped-shaped cavities, the cube side *l* is scaled according to Equation (2). Along with the options pertaining to the configuration files, DockBench 1.0 allows the user to optimize calculations performances by setting distributed computing and licenses management features. This functionality is designed to take advantage of multicore CPUs and makes a sophisticated use of semaphores, as implemented in GNU Parallel [24]. In details, all the jobs (docking runs) are classified and redirect to hardware resources according to two parameters: The total number of cores to be used—that is automatically detected by DockBench 1.0 but that can be edited by the user (*i.e.*, in case the calculations will run on a remote machine with a different cores number)—and the number of licenses available for commercial software packages. According to a classification based on the presence of licenses, the jobs are launched in different “traffic lines”: Protocols without license limits are redirected to the same traffic line, whereas to each licensed program a unique traffic line will be reserved. The number of licenses defines the width of the unique traffic lines, *i.e.*, how many jobs will simultaneously run for a given program. Therefore, the traffic lines reserved for licensed software packages will be subtracted from total number of cores and saturated by non-licensed jobs. For instance, on a workstation equipped with an eight core/threads CPU, DockBench1.0 (with default settings) will run simultaneously one GLIDE job, one GOLD job, and one MOE job. The remaining five cores will be saturated by the protocols not limited by licenses (AutoDock, AutoDock Vina, PLANTS, and rDOCK).

#### 2.1.3. Results Visualization

At the end of the docking simulations (Figure 1, green boxes), DockBench1.0 converts all the output files from formats specific to each docking software package to structure-data files (.sdf). A check is performed to detect any missing output, thus automatically identifying job failures. A summary of the chosen options as well as details on considered ligand-protein systems and tested protocols is reported in a format table on the GUI. For each structure-docking protocol pair, minimum (RMSDmin), maximum (RMSDmax) and average RMSD (RMSDave) values with respect to the X-ray binding mode are calculated (Equation (3), Experimental Section) and a text file summarizing all these results is produced (Figure 1, red boxes). These value are then used to compute the quality metrics [8] N^(RMSD < R)^ and the Protocol Score. At this stage, protocol and protein ranks are drafted and displayed in tabular format on the GUI according to the computed Protocol Score values. Protocol based ranks are derived by summing up the scores obtained by each protocol for all the considered protein structures. Protein based ranks are compiled by listing in descending orders the protein structures with higher sums of protocol scores. The GUI allow the user to shift from one rank to another according to which piece of information is considered more relevant.

#### 2.1.4. Plots Visualization

DockBench 1.0 provide the users with the possibility to graphically display the results as easy to interpret coloured maps. In the plot visualization tab, four plots depicting the RMSDmin, RMSDave, N^(RMSD < R)^, and Protocol Score trends are displayed. These graphs along with the above mentioned ranks are intended to guide the user in the selection of the best performing protocols as well as in the protein structure yielding more robust results for the subsequent VS jobs. In particular, each plot returns the list of tested protocols against the considered systems and display the analysed value (RMSDmin, RMSDave, N^(RMSD < R)^, or Protocol Score) with a colour code. To ease the interpretation of the results, colour codes have been devised so that blue spots identify the best results obtained for each value.

#### 2.1.5. Virtual Screening Settings

As anticipated, DockBench 1.0 offers the possibility to perform VS campaigns by selecting one or more of the previously evaluated docking protocols (Figure 1, orange box). The user is asked to upload a molecular database in .sdf format and has the possibility to automatically include the ligands used for the benchmark study (useful for enrichment analyses), and to define the number of posed to be returned for each ligand. Depending on the size of the loaded library and on the performance of the selected protocol detected during the benchmark procedure, an estimate of the time required to screen the whole library is provided. Similarly to the benchmark calculations, the VS scheme takes advantage of GNU parallel [24]. Calculation can be performed on a single workstation as well as on a cluster, by indicating the hostname and the number of cores to be used for each node. The jobs are monitored and in case of interruption, a restart input file is provided. To further speed up the calculations, the loaded library is splitted according to its size into more sdf files with an in-house python script implemented in the code. At the end of the VS procedure, the resulting conformers are merged and a global ranking is performed.

### 2.2. Case Study

**Figure 3:**
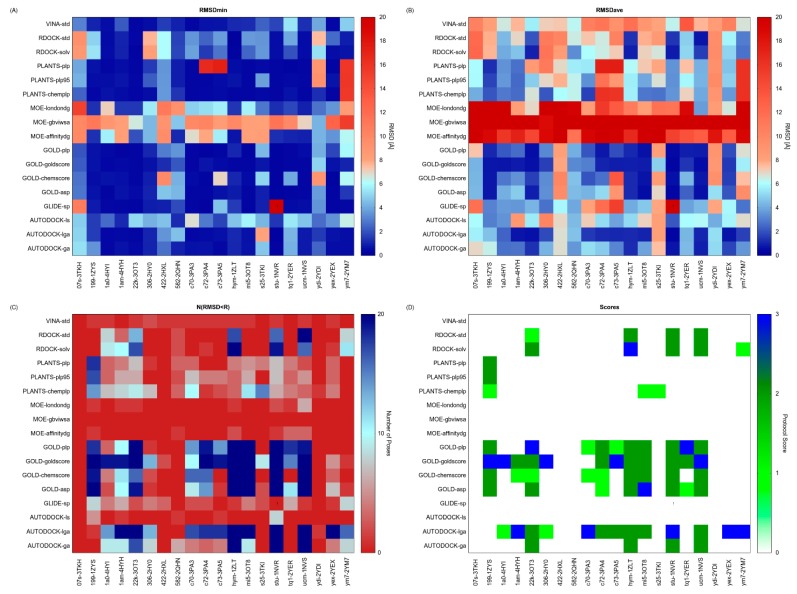
Results of the docking benchmark study on human checkpoint kinase 1. (**A**) Minimum RMSD values (RMSD_min_) returned by the tested docking protocol (y-values) for the considered X-ray structures (x-values); (**B**) Average RMSD values (RMSD_ave_); (**C**) Numbers of conformations returned by each docking protocol having a RMSD value lower than the X-ray structure resolution (N^(RSMD < R)^); (**D**) Protocol Score. RMSD is expressed in Å, whereas the Protocol Score on a 0–3 points scale. Values are rendered with a colour code, blue spots identify the best obtained results. Exclamation marks warn that an error occurred during docking calculations.

The results of our validation test are reported in Figure 3. The RMSDmin analysis highlights the protocols (VINA-std; GOLD-plp; GOLD-goldscore; GOLD-asp and AUTODOCK-ga) able to generate at least one pose that reproduces the X-ray observed binding mode with significant accuracy (Figure 3A). Some of these protocols, however, worsen their performances when RMSDave values are inspected (Figure 3B). Conversely, other protocols that accurately reproduced at least once the crystallographic pose for a given structure (GOLD-asp/c73-3PA5) show RMSDave values over the structures resolutions. By analysing the data in terms of N^(RMSD < R)^, it emerges that there are few protocols able to generate a high N^(RMSD < R)^ and that only in the 10% of the examined cases (32/340) all the conformations generated by the protocol have RMSD value below the structure resolution (N^(RMSD < R)^ = 20, blue spots in Figure 3C). The inspection of the Protocol Score results (Figure 3D) reveals that some protocols (RDOCK-solv, GOLD-plp, GOLD-goldscore, GOLD-asp and AUTODOCK-lga) generate the highest score for at least one protein structure. At a first glance, these results suggest that it is not possible to identify the best docking protocol for all the considered structures. Therefore, the selection of a proper protocol for subsequent docking simulations depends upon the selected protein structure. For instance, GOLD-goldscore could be used coupled to structures corresponding to PDB codes 1ZYS and 1NVS, whereas AUTODOCK-lga could be used in conjunction with the 1NVR structure. Overall, AUTODOCK-lga and GOLD-goldscore represent the protocols yielding the highest scores for a greater number of different proteins.

#### DockBench 1.0 Performances

To evaluate the performances of the distributed computing system we integrated in DockBench 1.0, we have tested the efficiency in the jobs management by DockBench 1.0 as compared with a traditional one by one job routine. In Table 2, the average execution time and the total calculation time for each protocol are reported. The docking calculation of the whole hChk1 case study (20 proteins; 17 Protocols) was achieved by the traditional routine in 16 h and 54 min. To complete the same task, DockBench 1.0 spent in 2 h 24 min, by using two licenses for GOLD, two licenses for GLIDE, two licenses for MOE and no license limit for the other software packages. It has to be pointed out that the DockBench 1.0 performances in this comparison were mainly affected by the low number of licenses used. A more reliable comparison has been drawn by running the same case study by using only non-licensed protocols (AutoDock, PLANTS, rDock, Vina). In this case, the traditional routine spent 11 h 13 min whereas DockBench 1.0 carried out the calculations in 27 min.

**Table 2:** Protocol performances for the hChk1 case study (20 proteins, 17 protocols). Time is expressed in seconds. Calculations were performed on a HP server equipped with four AMD Opteron6282 CPUs.

Abbreviation	Average Execution Time (s)	Total Time (s)
AUTODOCK-ga	973.5	20,445.3
AUTODOCK-lga	633.3	13,299.1
AUTODOCK-ls	7.45	156.58
GLIDE-sp	46.8	984.2
GOLD-asp	133.4	2801.8
GOLD-chemscore	136.2	2860.4
GOLD-goldscore	401.7	8436.5
GOLD-plp	98.6	2071.9
PLANTS-chemplp	61.4	1290
PLANTS-plp	23.3	958.1
PLANTS-plp95	16.6	348.3
MOE-affinitydg	17.6	352.5
MOE-londondg	18.4	368.4
MOE-gbviwsa	131.9	2638.8
RDOCK-std	20.0	426.2
RDOCK-solv	31.9	671.0
VINA-std	132.7	2786.7

## 3. Experimental Section

### 3.1. Computational Facilities

All computations were performed on a 200 cores cluster based on Ubuntu OS (14.04, 64 bit) and under the network file system (NFS) service. Performance timing of DockBench 1.0 was performed on a single HP ProLiant server DL585G7, equipped with four AMD Opteron Processor 6282 servers, for a total of 64 CPU cores.

### 3.2. DockBench 1.0 Platform

#### 3.2.1. Programming Languages and Software Dependencies

DockBench 1.0 is written in Python and patches several Bash scripts to launch and analyse molecular docking simulations. To integrate the MOE docking tool [22], in-house built Scientific Vector Language (SVL) scripts have been embedded in the code. DockBench 1.0 also integrates third party applications and the following packages are required to fully utilize the platform features: OpenBabel chemical toolbox 2.3.2 [14], GNU parallel 20130922 [24] and Gnuplot 4.6.

#### 3.2.2. Names Conventions

All the files generated by DockBench 1.0 are named according to the following scheme: “Ligand abbreviation—protein identifier—protocol abbreviation”. Ligands abbreviations correspond to the three letter codes assigned in the PDB files, whereas proteins identifiers are the corresponding PDB entries. Docking protocols abbreviations (Table 1) are named according to the following scheme: “Program name abbreviation-scoring function/search algorithm”.

#### 3.2.3. Implemented Docking Protocols and Standard Settings

In its current implementation, DockBench 1.0 handles the following docking software packages for a total of 17 different protocols (see Table 1 for more details): AutoDock 4.2.5.1 [15], AutoDock Vina1.1.2 [16], Glide 6.5 [17,18], GOLD 5.2 [25], MOE 2014.09 [22], PLANTS 1.2 [20], rDock [21]. Several common options among the different protocols have been set (Table 3).

**Table 3:** Common docking settings for the evaluated protocols.

Parameter	Value/Setting
Ligand input conformation	Structures generated by minimization
Ligand initial partial charges	Provided by the user
Water molecules	Excluded
Output	20 conformations (*customizable*)
RMSD threshold	1.0 Å (*customizable*)
Binding cavity centre (*Centroid*)	Ligand barycentre in X-ray structure
Binding cavity radius (*r*)	20 Å (*customizable*)
Grid spacing (for grid-based calculations)	0.375 Å
Refinement and re-scoring	Turned off

The coordinates of the binding cavity centre (centroid) are computed as the weighted centre of mass of all ligand atoms (Equation (1)):

(1) $$Centroid=\left(\frac{\sum_{i}^{n}\left(x_{i}*m_{i}\right)}{\sum_{i}^{n}m_{i}}\right),\left(\frac{\sum_{i}^{n}\left(y_{i}*m_{i}\right)}{\sum_{i}^{n}m_{i}}\right),\;\left(\frac{\sum_{i}^{n}\left(z_{i}*m_{i}\right)}{\sum_{i}^{n}m_{i}}\right)$$

To maintain similar cavity volumes for the protocols defining the binding cavity with a parallelepiped, we set cubes having similar volume to the sphere by scaling the side, *l*, according to Equation (2):

(2) $$l=\;\sqrt[{3}]{\frac{4\pi }{3}}\;r$$

Moreover, at variance with the previously published procedure [8], a pre-processing step of the input conformations has been implemented to avoid biases arising from ligand input conformation. The input structures are therefore minimized with the minimize tool [14], using the conjugate gradient algorithm and a maximum of 2500 steps to reach convergence criteria of 1e-16 based on the MMFF94 force field [26]. Finally, RMSD values with respect to the co-crystallized ligands are calculated as reported in Equation (3) with an in-house built Python script. Given two sets of *n* heavy atoms **a** and **b**:

(3) $$RMSD\left(a,b\right)=\;\sqrt{\frac{1}{n}\overset{n}{\underset{i=1}{\sum }}\left({\left(a_{ix}-b_{ix}\right)}^{2}+{\left(a_{iy}-b_{iy}\right)}^{2}+{\left(a_{iz}-b_{iz}\right)}^{2}\right)}$$

### 3.3. Case Study Input Files Preparation

#### 3.3.1. Protein Structures

Among the 108 available X-ray structures for the hChk1, the following 20 ligand-protein complexes were selected for the docking benchmark (PDB IDs): 3TKH [27], 3TKI [27], 1ZYS [28], 4HYI [29], 4HYH [29], 3OT3 [30], 2HY0 [31], 2HXL [31], 2QHN [32], 3PA3 [33], 3PA4 [33], 3PA5 [33], 1ZLT [34], 3OT8 [35], 1NVR [36], 1NVS [36], 2YEX [37], 2YER [37], 2YDI [38], 2YM7 [39]. The structures were retrieved from the RCSB PDB database[13] and selected on the basis of their X-ray resolution (R, selection criterion = R < 1.8 Å). Before the preparation procedure, all the proteins were aligned and superimposed to a selected reference structure. Crystallization solvent and ions were removed, whereas water molecules and co-crystallized ligands were retained for the hydrogen atoms assignment step and then removed. Ionization states and hydrogen positions were assigned with the ‘Protonate-3D’ tool [40], as implemented in the Molecular Operating Environment (MOE, version 2014.09) suite [22]. Then, the structures were subjected to energy minimization with Amber99 force field [41], by keeping the heavy atoms fixed at their positions. Finally, ligand and water molecules were removed and protein atoms partial charges computed with the Amber99 force field [41].

#### 3.2.2. Ligand Structures

Co-crystallized ligands were extracted from the corresponding crystallographic complex and checked for errors. Hydrogen atoms were added and the protonation state (pH: 7.4) was assigned. Partial charges on ligands atoms were computed on the basis of the PM3/ESP semiempirical Hamiltonian [42,43]. The structures have been then subjected to the ligand preparation procedure of the DockBench 1.0 platform. A full list of ligands considered in this study along with their structures and names is reported in Table 4.

**Table 4:** List of ligand structures considered in this study.

Structure	IUPAC Name	Ligand Abbreviation
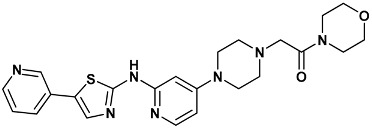	1-morpholin-4-yl-2-[4-[2-[(5-pyridin-3-yl-1,3-thiazol-2-yl)amino]pyridin-4-yl]piperazin-1-yl]ethanone	07s-3TKH
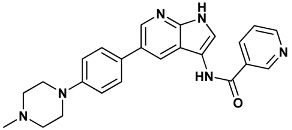	*N*-{5-[4-(4-methylpiperazin-1-yl)phenyl]-1*H*-pyrrolo[2,3-*b*]pyridin-3-yl}pyridine-3-carboxamide	199-1ZYS
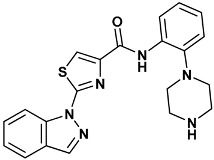	2-indazol-1-yl-*N*-(2-piperazin-1-ylphenyl)-1,3-thiazole-4-carboxamide	1a0-4HYI
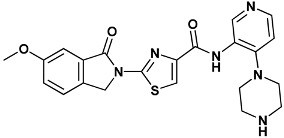	2-(6-methoxy-1-oxoisoindolin-2-yl)-*N*-(4-(piperazin-1-yl)pyridin-3-yl)thiazole-4-carboxamide	1am-4HYH
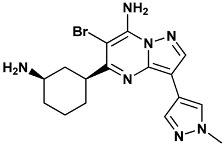	5-[(1*R*,3*S*)-3-azanylcyclohexyl]-6-bromo-3-(1-methylpyrazol-4-yl)pyrazolo[1,5-*a*]pyrimidin-7-amine	22k-3OT3
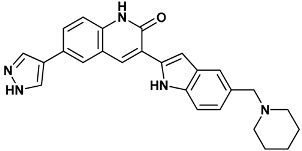	3-[5-(piperidin-1-ylmethyl)-1*H*-indol-2-yl]-6-(1*H*-pyrazol-4-yl)-1*H*-quinolin-2-one	306-2HY0
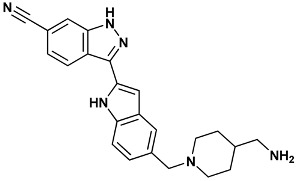	3-[5-[[4-(aminomethyl)piperidin-1-yl]methyl]-1*H*-indol-2-yl]-1*H*-indazole-6-carbonitrile	422-2HXL
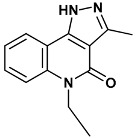	5-ethyl-3-methyl-1*H*-pyrazolo[4,5-*c*]quinolin-4-one	582-2QHN
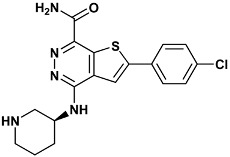	2-(4-chlorophenyl)-4-[[(3*S*)-piperidin-3-yl]amino]thieno[2,3-*d*]pyridazine-7-carboxamide	c70-3PA3
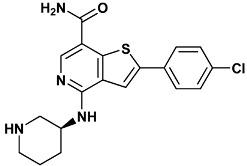	2-(4-chlorophenyl)-4-[[(3*S*)-piperidin-3-yl]amino]thieno[3,2-*c*]pyridine-7-carboxamide	c72-3PA4
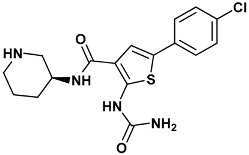	2-(aminocarbonylamino)-5-(4-chlorophenyl)-*N*-[(3*S*)-piperidin-3-yl]thiophene-3-carboxamide	c73-3PA5
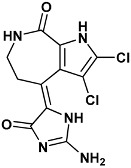	(4*Z*)-4-(2-amino-5-oxo-3*H*-imidazol-4-ylidene)-2,3-dichloro-1,5,6,7-tetrahydropyrrolo[2,3-*c*]azepin-8-one	hym-1ZLT
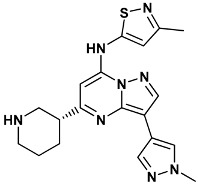	3-(1-methyl-1*H*-pyrazol-4-yl)-*N*-(3-methyl-1,2-thiazol-5-yl)-5-[(3*R*)-piperidin-3-yl]pyrazolo[1,5-*a*]pyrimidin-7-amine	mi5-3OT8
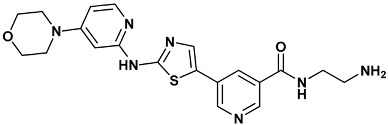	*N*-(2-azanylethyl)-5-[2-[(4-morpholin-4-ylpyridin-2-yl)amino]-1,3-thiazol-5-yl]pyridine-3-carboxamide	s25-3TKI
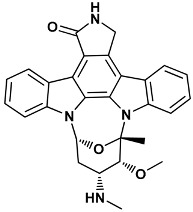	(5*S*,6*R*,7*R*,9*R*)-6-methoxy-5-methyl-7-(methylamino)-6,7,8,9,15,16-hexahydro-17-oxa-4*b*,9*a*,15-triaza-5,9 methanodibenzo[*b*,*h*]cyclonona[*jkl*]cyclopenta[e-as-indacen-14(5*H*)-one	stu-1NVR
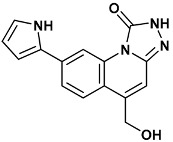	5-(hydroxymethyl)-8-(1*H*-pyrrol-2-yl)[1,2,4]triazolo[4,3-*a*]quinolin-1(2*H*)-one	tq1-2YER
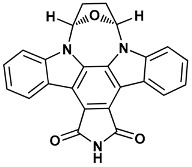	(5*R*,8*S*)-5,6,7,8-tetrahydro-13*H*-5,8-epoxy-4*b*,8*a*,14-triazadibenzo[*b*,*h*]cycloocta[1,2,3,4-*jkl*]cyclopenta[*e*]-as-indacene-13,15(14*H*)-dione	ucm-1NVS
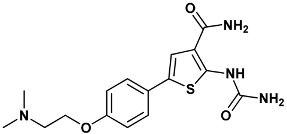	2-(carbamoylamino)-5-{4-[2-(dimethylamino)ethoxy]phenyl}thiophene-3-carboxamide	ydi-2YDI
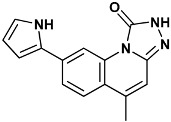	5-methyl-8-(1*H*-pyrrol-2-yl)-2*H*-[1,2,4]triazolo[4,3-*a*]quinolin-1-one	Yex-2YEX
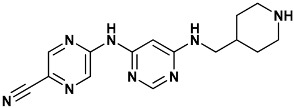	5-((6-((piperidin-4-ylmethyl)amino)pyrimidin-4-yl)amino)pyrazine-2-carbonitrile	ym7-2YM7

## 4. Conclusions

We have introduced here DockBench 1.0, a platform available free of charge that fully automates the pipeline from docking benchmarks to VS campaigns setups. Currently, DockBench 1.0 implements seven different docking software packages (including commercial and freely available ones) and provides the user with the possibility to test up to seventeen protocols. The platform has been devised with the aim to minimize the user’s required expertise by overcoming the main issues related to docking benchmark procedures: The management of input/output formats and the time required in running, merging and comparing the results arising from different software packages. To this aim, a GUI guides the user step-by-step throughout all the stages from docking protocol assessment to VS of large chemical libraries. The outputs of the different software packages are returned in a unique format and are analysed with a standardized procedure to avoid biases. The distributed computing philosophy based on GNU parallel semaphores has been integrated in the platform, thus allowing the users to speeds up the calculations while cleverly using the available resources. As validation case, we have reported on the benchmark study of 20 hChk1 structures by testing all the protocols available in the platform. DockBench 1.0 is available free of charge and can be requested at the project web page [12].
